# Comprehensive Approach to Distinguish Patients with Solid Tumors from Healthy Controls by Combining Androgen Receptor Mutation p.H875Y with Cell-Free DNA Methylation and Circulating miRNAs

**DOI:** 10.3390/cancers14020462

**Published:** 2022-01-17

**Authors:** Elena Tomeva, Olivier J. Switzeny, Clemens Heitzinger, Berit Hippe, Alexander G. Haslberger

**Affiliations:** 1 HealthBioCare GmbH, A-1090 Vienna, Austria; et@healthbiocare.at (E.T.); switzeny@healthbiocare.at (O.J.S.); bh@healthbiocare.at (B.H.); 2 Center for Artificial Intelligence and Machine Learning (CAIML), TU Wien, A-1040 Vienna, Austria; clemens.heitzinger@tuwien.ac.at; 3 Department of Nutritional Sciences, University of Vienna, A-1090 Vienna, Austria

**Keywords:** cancer, classification, liquid-biopsy, microRNA, cell-free DNA, biomarker, methylation

## Abstract

**Simple Summary:**

Blood-based tests for cancer detection are minimally invasive and could be useful for screening asymptomatic patients and high-risk populations. Since a single molecular biomarker is usually insufficient for an accurate diagnosis, we developed a multi-analyte liquid biopsy-based classification model to distinguish cancer patients from healthy subjects. The combination of cell-free DNA mutations, miRNAs, and cell-free DNA methylation markers improved the model’s performance. Moreover, we demonstrated that the androgen receptor mutation p.H875Y is not only relevant in prostate cancer but had a strong predictive value for colorectal, bladder, and breast cancer. Our results, although preliminary, showed that a single liquid biopsy test could detect multiple cancer types simultaneously.

**Abstract:**

Liquid biopsy-based tests emerge progressively as an important tool for cancer diagnostics and management. Currently, researchers focus on a single biomarker type and one tumor entity. This study aimed to create a multi-analyte liquid biopsy test for the simultaneous detection of several solid cancers. For this purpose, we analyzed cell-free DNA (cfDNA) mutations and methylation, as well as circulating miRNAs (miRNAs) in plasma samples from 97 patients with cancer (20 bladder, 9 brain, 30 breast, 28 colorectal, 29 lung, 19 ovarian, 12 pancreas, 27 prostate, 23 stomach) and 15 healthy controls via real-time qPCR. Androgen receptor p.H875Y mutation (*AR*) was detected for the first time in bladder, lung, stomach, ovarian, brain, and pancreas cancer, all together in 51.3% of all cancer samples and in none of the healthy controls. A discriminant function model, comprising cfDNA mutations (COSM10758, COSM18561), cfDNA methylation markers (*MLH1*, *MDR1*, *GATA5*, *SFN*) and miRNAs (miR-17-5p, miR-20a-5p, miR-21-5p, miR-26a-5p, miR-27a-3p, miR-29c-3p, miR-92a-3p, miR-101-3p, miR-133a-3p, miR-148b-3p, miR-155-5p, miR-195-5p) could further classify healthy and tumor samples with 95.4% accuracy, 97.9% sensitivity, 80% specificity. This multi-analyte liquid biopsy-based test may help improve the simultaneous detection of several cancer types and underlines the importance of combining genetic and epigenetic biomarkers.

## 1. Introduction

Cancer is mostly a manageable disease as long as it is diagnosed and treated before metastasis has begun. In most cases, higher-grade cancer evolves from lower-grade cancer. Thus, early tumor detection could increase the chances of successful treatment. In this way, carcinomas could be identified at an early stage when they can still be surgically removed and cured [1,2]. Therefore, scientists are focusing on discovering biomarkers for early cancer detection.

Liquid biopsy is a rapidly developing tool for assessing biomarkers shed from difficult to access tissue in easily sampled bodily fluids, such as urine, blood, saliva, sweat, feces, and tears [3,4]. Such minimally invasive blood-based tests could be useful for screening asymptomatic patients and high-risk populations. Liquid biopsy-based tests have been not only successfully applied in disease screening [5] but tend to have even higher compliance in comparison to other standard procedures such as FIT (fecal immunochemical test) [6].

Cell-free DNA (cfDNA) and circulating miRNA from apoptotic, necrotic, or viable tumor cells are released into the bloodstream. Tumor-derived cfDNA harbors somatic mutations originating from the tumor and comprises tissue-specific DNA methylation patterns; thus, methylation can indicate tumor location [7,8]. Hence an organ-specific epigenetic pattern is measurable in the circulation [9]. Since many tumors originating from different tissues share identical SNPs [10], epigenetic information adds a tissue-specific data layer [11]. 

Given that epigenetic alterations occur early in carcinogenesis, cfDNA methylation markers and miRNAs could be early cancer predictors. Genome-wide miRNA expression profiling by miRNA sequencing led to finding useful biomarkers for the early diagnosis of various cancers [12]. Moreover, a neural network model including only serum miRNA could successfully differentiate between cancer, non-invasive neoplasms, and healthy controls and suggests that detection of pre-metastatic disease in serum is possible [13]. 

Nevertheless, the heterogeneous phenotype of many diseases leads to variability in biomarker expression across individuals. Usually, a single molecular biomarker is not sufficient for an accurate diagnosis of cancer [14,15,16]. When using only mutation biomarkers, after a certain number of markers is reached, adding additional mutation biomarkers would fail to improve the sensitivity of the test and increase the false positive rate [3]. Thus, a multi-analyte combined test could address this challenge. A combination of more than one analyte has been found to improve the performance of liquid biopsy-based detection tests [17,18,19].

These methods’ huge amount of data needs automated data processing to deliver clinically relevant information. They range from simple approaches, such as logistic regression and support vector machines, to complex artificial neural networks with many hidden layers [20]. 

Despite the numerous aforementioned advantages of liquid biopsy, this theoretically simpler approach to longitudinal disease monitoring, as opposed to tissue biopsy, is still not routinely applied in cancer management. The low amount of cell-free DNA, the limited sensitivity, and specificity remain a challenge, and the clinical applicability has yet to be established [21].

Eventually, given the potential of this approach, an accurate, simple, and minimally invasive pan-cancer screening test could ensure wide use, especially in a high-risk population. This approach could reach more patients more rapidly since they would be screened for several cancer entities. Ideally, this test would be able to identify the tissue of origin if a malignancy is detected. In this study, our objective was to determine whether miRNAs, cfDNA mutations, and cfDNA methylation can be combined to differ samples from subjects with bladder, brain, breast, colorectal, lung, ovarian, prostate, stomach, and pancreatic cancers and samples from cancer-free subjects, consequently creating a multi-analyte liquid biopsy-based test. To our best knowledge, we are the first to combine mutations, miRNAs, and DNA methylation markers to test several tumor entities.

## 2. Materials and Methods

### 2.1. Study Population

The study was approved by the local ethics committees and carried out according to the current EU directives. All study subjects were recruited by Fidelis Research AD, Bulgaria, and included after written informed consent. Study participants had to be male or female above the age of 25 without a previously treated cancer. Plasma was obtained from a total of 205 patients with stage I, II, or III cancer prior to cancer therapy and a control group (*n* = 15) of subjects with no evidence of malignancies. Nonetheless, 7 subjects of the control group had one of the following conditions at the sample collection point: pulmonary fibrosis, renal cyst, hemorrhoidal disease, dyspepsia, peritonsillar phlegmon, or endometrial polyp. Cancer types included in the study were liver, lung, pancreas, colorectal cancer, prostate, ovarian, breast, stomach, bladder, and brain cancer. The clinical data of these study groups are summarized in Table 1.

### 2.2. Sample Collection and Liquid Biopsy 

Peripheral venous blood samples were collected prior to surgery and therapy in K-2 EDTA vacutainers. Subsequently, plasma was separated via double centrifugation as described previously [22]. Whole blood samples were processed within one hour after the blood draw. Briefly, blood samples were centrifuged at 2000× *g* for 10 min at 4 °C, followed by centrifugation of the supernatant at 16,000× *g* for 10 min at 4 °C. The prepared plasma samples were stored at −80 °C until shipment. All samples were shipped frozen (−20 °C) on dry ice and stored temporarily at −20 °C upon arrival.

### 2.3. Cell-Free DNA Extraction, Processing, and Analysis

Cell-free DNA (cfDNA) was isolated with MagMAX™ Cell-Free DNA Isolation Kit (ThermoFisher Scientific, Waltham, MA, USA) using KingFisher™ Duo Prime Magnetic Particle Processor (ThermoFisher Scientific, Waltham, MA, USA) according to the user guidelines. CfDNA was isolated from 4 mL plasma and eluted with elution solution in a final volume of 80 µL. The purified cfDNA samples were stored at −20 °C until further analysis. DNA was quantified using the dsDNA HS Assay Kit on Qubit 4 Fluorometer (Invitrogen, ThermoFisher Scientific, Waltham, MA, USA) according to the standard kit protocol. 

### 2.4. Mutation Analysis

Prior quantitative real-time PCR (qPCR) analysis of cfDNA mutations, a blunt end ligation-mediated whole genome amplification (BL-WGA) was carried out as described previously [23]. Briefly, 4.45 μL purified cfDNA (~ 0.5 to 20 ng total/4 ng average) was blunted with 0.3 U of T4 DNA polymerase (New England Biolabs (NEB), Frankfurt am Main, Germany) in 0.5 μL of 10× T4 DNA ligase buffer (NEB), supplemented with dNTPs (ThermoFisher) at a final concentration of 100 µmol/L at 12 °C for 15 min. The reaction was then inactivated at 75 °C for 20 min. The blunted DNA was then ligated with 500 U of T4 DNA ligase (NEB) at room temperature for 2 h and subsequently inactivated at 65 °C for 10 min. Afterward, the sample was denatured at 95 °C for 3 min and then rapidly cooled on ice for 3–5 min in a total volume of 10 µL, containing 1 µL EquiPhi29 DNA Polymerase Reaction Buffer 10× (ThermoFisher), 2.55 µL nuclease-free water (nfw) and 100 µM exo-resistant random primer (ThermoFisher). Next, the sample was amplified at 45 °C for 3 h using 10 U of EquiPhi29 DNA Polymerase (ThermoFisher), 1 mM DTT (ThermoFisher), 1 mM dNTPs (ThermoFisher), 0.02 U pyrophosphatase (ThermoFisher), 1.5 µL EquiPhi29 DNA Polymerase Reaction Buffer 10× and 4.5 µL nfw in a total volume of 20 µL. Finally, the reaction was stopped by heat-inactivation at 65 °C for 10 min. The samples were quantified via Qubit 4 Fluorometer using dsDNA BR Assay Kit (ThermoFisher).

The BL-WGA cfDNA (10–20 ng pro reaction) was then used for the mutational analysis with TaqMan™ Mutation Detection Assays (ThermoFisher). The array is designed to analyze 75 cancer-specific mutations in 21 genes and consists of a Reference Assay for the amplification of a mutation-free and polymorphism-free region of the target gene in addition to the Mutation Assay. Namely, the genes are *AKT1*, *APC*, *AR*, *BRAF*, *CTNNB1*, *EGFR*, *ERBB2*, *ESR1*, *FBXW7*, *FGFR3*, *GNAS*, *HRAS*, *IDH1*, *KRAS*, *MED12*, *NRAS*, *PIK3CA*, *SMAD4*, *TERT*, *TP53*, and *VHL* (Supplementary Table S1). ΔCt values for the detection of mutations were established for each gene and defined as:

ΔCt=Ct(Mutation Assay)−Ct(Reference Assay).

The presence of a mutation in a sample was determined upon an assay-specific cutoff point (Supplementary Table S1). The DNA mutation screening was performed on a QuantStudio 3 Real-Time PCR System (Applied Biosystems, ThermoFisher).

### 2.5. Methylation Analysis

For methylation analysis, 70 µL of the purified cfDNA was divided into two fractions—one containing methylated cfDNA and one containing unmethylated cfDNA, using MethylMiner™ Methylated DNA Enrichment Kit (Invitrogen, ThermoFisher). This method is based on the binding of methylated DNA to MBD2 protein which is coupled to magnetic beads. The methylated fragments can then be eluted as a single enriched fraction with a high salt concentration solution (NaCl), thereby separating methylated (Me cfDNA) from unmethylated cfDNA (UnME cfDNA). Both fractions were subsequently quantified via real-time qPCR for 12 different cancer-relevant genetic regions (*SEPT9*, *MLH1*, *MGMT*, *GATA5*, *GSTP1*, *SFN*, *MDR1*, *VIM*, *SHOX2*, *ALKBH3*, *APC*, *RASSF1A*). Then, 2 µL of each fraction of cfDNA was amplified using a custom-designed primer (150 nM, Supplementary Table S2) and GoTaq^®^ qPCR Master Mix (Promega, US) in a final volume of 10µL on QuantStudio 3 (ThermoFisher). The methylation level for each region was calculated using the following formula:

$$CfDNA\;methylation\;\%=100-(100/\left(1+2^{-Ct\mathrm{Me}\;\mathrm{cfDNA}\;-Ct\mathrm{UnMe}\;\mathrm{cfDNA}}\right).$$

### 2.6. RNA Extraction, Processing, and microRNA Analysis

Total RNA was isolated from 100 µL plasma with MagMAX™ mirVana™ Total RNA Isolation Kit (ThermoFisher Scientific, Waltham, MA, USA) using KingFisher™ Duo Prime Magnetic Particle Processor (ThermoFisher Scientific, Waltham, MA, USA) according to the user guidelines. Spike-in miRNA *C. elegans* 39 was added during the RNA purification at a concentration of 15 fmol per sample. Total RNA was eluted with elution buffer in final volumes of 50 μL, and samples were stored at −20 °C until further analysis.

After RNA purification, miRNA was transcribed into cDNA using the TaqMan™ Advanced miRNA cDNA Synthesis Kit (ThermoFisher). A 1:10 dilution of the cDNA was taken for the analysis of 48 miRNAs (*C. elegans* spike-in control, Supplementary Table S3) using prespotted Taqman adv. miRNA 96 well plates (ThermoFisher) on a QuantStudio 3 Real-Time PCR System (ThermoFisher) in a final reaction volume of 10µL. For data normalization global mean of all analyzed miRNAs were used as previously described [24]. 

### 2.7. Statistical Analysis

One-way ANOVA for continuous variables and χ2 test and cross-tabulation for categorical variables were used to analyze the characteristics of the subjects. A *t*-test of independent samples was performed to compare the mutational burden between cancer-free subjects and cancer patients. The correlation between cell-free DNA concentration and cancer stage was analyzed with a Spearman’s ρ rank coefficient test. miRNA expression values were standardized by converting them to Z-scores. A one-way ANOVA was carried out to determine whether miRNAs are differently expressed or cfDNA methylation varies across the test groups. A χ^2^ automatic interaction detection decision tree model (CHAID) was used to split the samples into subsets. The diagnostic potential of cfDNA mutations, cfDNA methylation markers, and miRNAs was analyzed in discriminant function analyses (DA) with a leave-one-out cross-validation. The performance of these DAs was further estimated by a receiver operating characteristic (ROC) analysis and area under the curve (AUC). Statistical analyses were carried out in IBM^®^ SPSS^®^ Statistics 20 Software. 

### 2.8. Identification of Candidate Biomarkers

For the identification of the potential diagnostic markers, the correlation matrix of all variables was calculated (Supplementary Figure S1). Firstly, all variables with missing values were excluded from the analysis. Further, continuous variables (miRNA level and cfDNA methylation percentage) were dichotomized upon an automatically defined threshold value.

Subsequently, for each cancer type, the correlations of each cancer type with each biomarker were calculated and sorted by their absolute values (Supplementary Figure S2). Since many measured variables compared to a relatively small sample size tend to produce spurious correlations, a subset of the best biomarkers with the highest correlation (by absolute value) for each cancer type was chosen for further tests.

Then, all combinations of these best biomarkers were tested regarding their importance to predict a particular tumor type versus the healthy control group. Variables with redundant information were eliminated based on a covariance matrix to further alleviate the effects of overfitting. Thus, superfluous biomarkers that yield no improvement concerning the classification performance of each cancer type were excluded. In order to do so, a score was defined, where false negatives are discouraged by a factor of two compared to false positives. All computations were carried out in R version 4.1.2.

## 3. Results

### 3.1. Patient Characteristics

A total of 205 cancer and 15 cancer-free plasma samples were collected. One of the cancer samples was excluded since the patient was diagnosed with stage 4 ovarian cancer after plasma collection. We received only seven plasma samples from patients with liver cancer, making the size of this sample group too small to yield any meaningful results and thereby was excluded from the statistical data analysis. Hence, a total of 212 samples were analyzed for mutations, miRNAs, and DNA methylation. Patient characteristics are summarized in Table 1.

One-way ANOVA test showed significant differences between the age of the healthy control group and bladder cancer (*p* < 0.001), CRC (*p* < 0.05), prostate cancer (*p* < 0.05), and stomach cancer (*p* < 0.005). The BMI of the subjects with ovarian cancer differed significantly from the BMI of the patients with CRC (*p* < 0.05) and stomach cancer (*p* < 0.001).

### 3.2. Plasma cfDNA Levels

Mean plasma cfDNA levels did not significantly differ between cancer patients and healthy controls (mean cfDNA plasma levels of cancer patients = 1.514 ng/µL, mean cfDNA plasma levels of healthy subjects = 0.557 ng/µL, *p* = 0.439). However, a significant correlation between cfDNA concentration and cancer stage (R = 0.225, *p* < 0.001, *n* = 212) was observed. Mean cfDNA plasma levels irrespective of cancer type were 0.435 ng/µL for stage I (*n* = 39), 1.091 ng/µL for stage II (*n* = 81), and 2.506 ng/µL for stage III (*n* = 77).

### 3.3. Plasma cfDNA Mutation Detection

Targeted mutation analysis was implemented to investigate 75 alterations such as nucleotides insertions and substitutions (Supplementary Table S1), referred to as mutations. Among the 197 patients with tumors, at least one mutation was detected in 187 patients (94.9%). In 8 out of the 15 healthy control samples, at least one mutation was detected in *CTNNB1* (COSM5663), *EGFR* (COSM6224), *FGFR3* (COSM718), *KRAS* (COSM517), *PIK3CA* (COSM760), *TP53* (COSM10662, COSM6549, COSM10690, COSM10863). The six most frequently detected mutations among all samples were in *AR* (COSM238555, *n* = 101, 48%), *EGFR* (COSM6224, *n*= 97, 46%), *TP53* (COSM10758, *n* = 77, 36%), *FGFR3* (COSM718, *n* = 64, 30%), *TERT* (COSM1716559, *n* = 46, 22%), *APC* (COSM18561, *n* = 45, 21%) (Supplementary Table S4). The mutations COSM5677 (*CTNNB1*), COSM6223 (*EGFR*), COSM22932 (*FBXW7*), COSM483 (*HRAS*), COSM499 (*HRAS*), COSM518 (*KRAS*), COSM10779 (*TP53*) were not detected in any sample and were therefore excluded from the analysis. All of the analyzed cfDNA alterations and their frequencies in this study population are listed in Supplementary Table S4. 

A significant difference in the mutation burden of cfDNA between healthy subjects and cancer patients was prominent (*p* < 0.001, mean difference = 4.819, std error = 0.463, 95% CI 3.896–5.741). Cancer patients had 6.15, while the control group had 1.33 mutations on average.

### 3.4. Androgen Receptor p.H875Y Mutation

A total of 101 (51.3%) of all cancer patients had a p.H875Y mutation in the androgen receptor gene (*AR*, COSM238555) (Supplementary Table S4). This mutation was detected in the plasma of subjects with CRC (85.71%), bladder (80%), prostate (66.67%), and breast (60%) cancer samples, and none of the healthy controls. The percentages of *AR* mutation-positive patients (AR+) for lung, stomach, ovarian, brain, and pancreas cancer were 48.28, 26.09, 20, 11.11, and 8.33, respectively. Interestingly, cancer samples with an *AR* mutation (*n* = 101) had an overall higher total mutational burden than samples without an *AR* mutation (AR–, *n* = 96), 7.5 and 4.8 mutations on average, respectively (*p* < 0.001).

### 3.5. Plasma cfDNA Methylation

**Figure 1:**
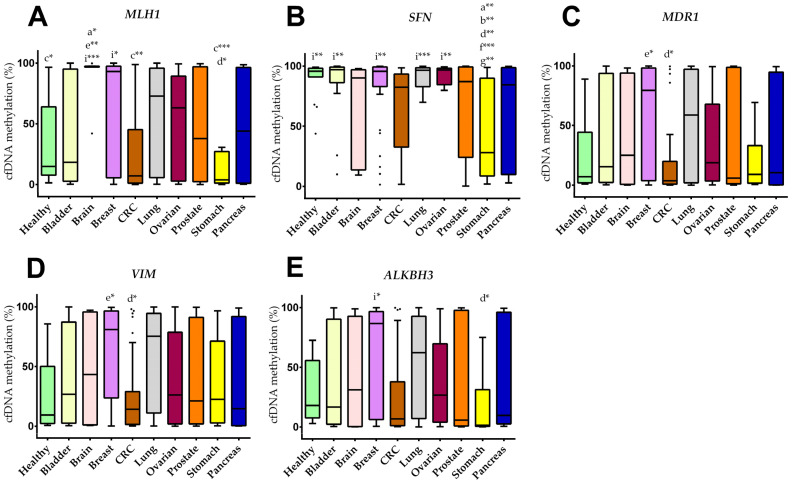
Cancer type specific cfDNA methylation levels. (**A**–**E**) Boxplots of cfDNA methylation for (**A**) *MLH1*, (**B**) *SFN*, (**C**) *MDR1*, (**D**) *VIM*, (**E**) *ALKBH3*. Boxes are the 25th to 75th percentile; the line is the median, and whiskers are 1.5× IQR. *p*-values are showed as * *p* < 0.05; ** *p* < 0.005; *** *p* < 0.001. Lower case letters indicate the group with significantly different cfDNA methylation levels: a healthy, b bladder, c brain, d breast, e CRC, f lung, g ovarian, i stomach.

The methylation levels (m%) of 12 different cancer-relevant genetic regions (*SEPT9*, *MLH1*, *MGMT*, *GATA5*, *GSTP1*, *SFN*, *MDR1*, *VIM*, *SHOX2*, *ALKBH3*, *APC*, *RASSF1A*) were analyzed for all 212 samples. The cfDNA methylation levels for *MLH1*, *SFN*, *MDR1*, *VIM*, and *ALKBH3* of all groups are shown in Figure 1. No significant differences for *SEPT9*, *MGMT*, *GATA5*, *GSTP1*, *SHOX2*, *APC*, and *RASSF1* between the different study groups were observed. The data for all analyzed genetic regions are provided in a heatmap in Supplementary Figure S3.

### 3.6. Identification of Differently Expressed Circulating miRNAs

**Figure 2:**
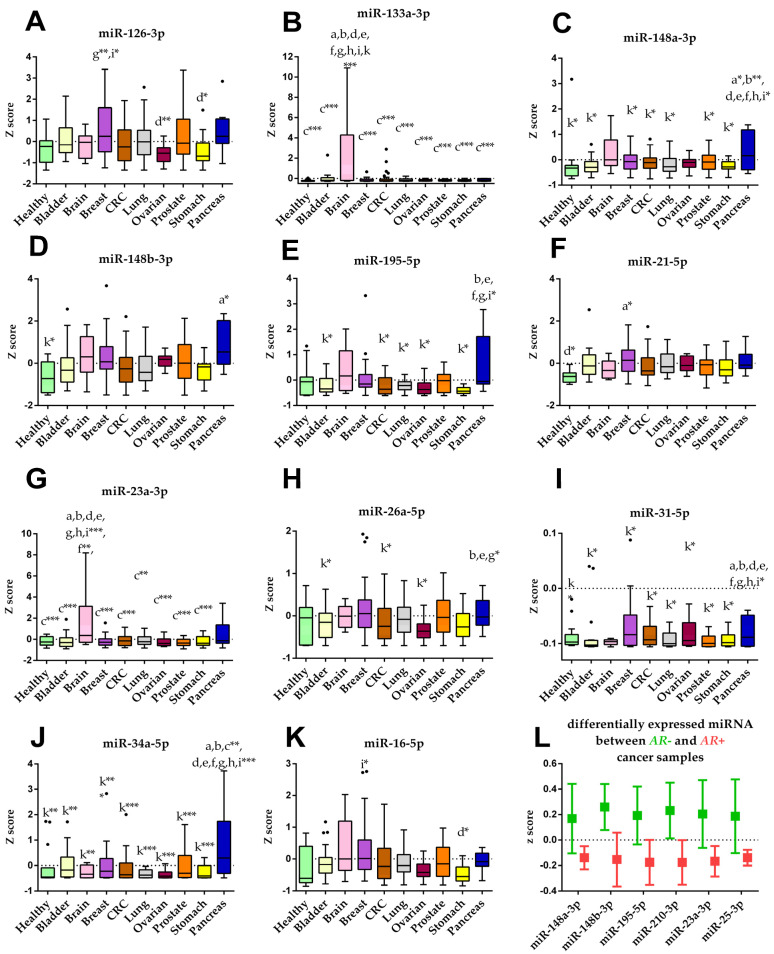
Cancer type specific miRNA expression levels. (**A**–**K**) Boxplots of the miRNAs deregulated between the different cancer types and control group (healthy). Boxes are the 25th to 75th percentile; the line is the median, and whiskers are 1.5× IQR. Lower case letters indicate the group with significantly different miRNA levels: a healthy, b bladder, c brain, d breast, e CRC, f lung, g ovarian, h prostate, i stomach, k pancreas. (**L**) Grouped plot of the differentially expressed miRNAs between cancer samples with (AR+) and without (AR–) p.H875Y androgen receptor mutation. The line is the mean value, and whiskers are 95% CI. *p*-values are showed as * *p* < 0.05; ** *p* < 0.005; *** *p* < 0.001.

Among the 47 analyzed miRNAs, four were under the detection limit for the reference sample (miRNAs 30a-5p, 218-5p, 1225-3p, 203a-3p); therefore, they were excluded from the analysis. A heatmap was generated for the remaining 43 miRNAs (Supplementary Figure S4). After computing the Z-scores for the miRNA expression data, a differential analysis was conducted, and significantly deregulated miRNAs are depicted in Figure 2. MiRNAs 133a-3p and 23a-3p were significantly up-regulated on subjects with brain (Figure 2B and Figure 2G respectively). MiR-148a-3p was significantly elevated in subjects with pancreas cancer compared to all groups except brain and ovarian cancer (Figure 2C). Additionally, higher levels of miR-34a-5p for subjects with pancreas cancer were observed (Figure 2J). Furthermore, miR-31-5p in pancreas cancer was down-regulated compared to breast and ovarian cancer and up-regulated compared to the bladder, CRC, lung, prostate, stomach cancer, and the control group (Figure 2I). Interestingly, cancer samples with an *AR* mutation (AR+, *n* = 101) showed significantly lower levels of miRNAs 148a-3p, 148b-3p, 195-5p, 210-3p, 23a-3p, 25-3p when compared to samples without an *AR* mutation (AR–, *n* = 96) (Figure 2L).

### 3.7. Identification of Cancer Type Specific Biomarkers 

A search algorithm for the most predictive cfDNA mutations, miRNAs, and cfDNA methylation markers for each cancer type was derived and implemented, and variables with redundant information were eliminated based on a score that discourages false positives. The correlations for each cancer type are shown in Supplementary Figure S2A–I. Depending on the cancer type, three to four biomarkers per cancer type showed the highest correlations compared to the healthy control (Table 2).

### 3.8. Classification of Tumor Samples

Firstly, samples were split into two groups (χ^2^ 14.688, *p* < 0.001): samples with an *AR* mutation (AR+, *n* = 101) and samples without an *AR* mutation (AR–, *n* = 111). The AR+ group consisted only of tumor samples since no *AR* mutation was detected in the control group. However, the AR– group contained the healthy controls (*n* = 15) and tumor samples (*n* = 96); therefore, no further classification of these groups was possible based only on *AR* mutation. In order to separate healthy from tumor samples in the AR– group, several discriminant function analyses with a leave-one-out cross-validation were carried out, including different sets of biomarkers, not including *AR* mutation. The sets of biomarkers were as follows: discriminant analysis 1 (DA1) incorporated all measured targets; discriminant analysis 2 (DA2) only cfDNA mutations; discriminant analysis 3 (DA3) only cfDNA methylation; discriminant analysis 4 (DA4) only miRNAs; discriminant analysis 5 (DA5) included the biomarkers with highest correlations identified through the correlation matrixes (Table 3).

**Figure 3:**
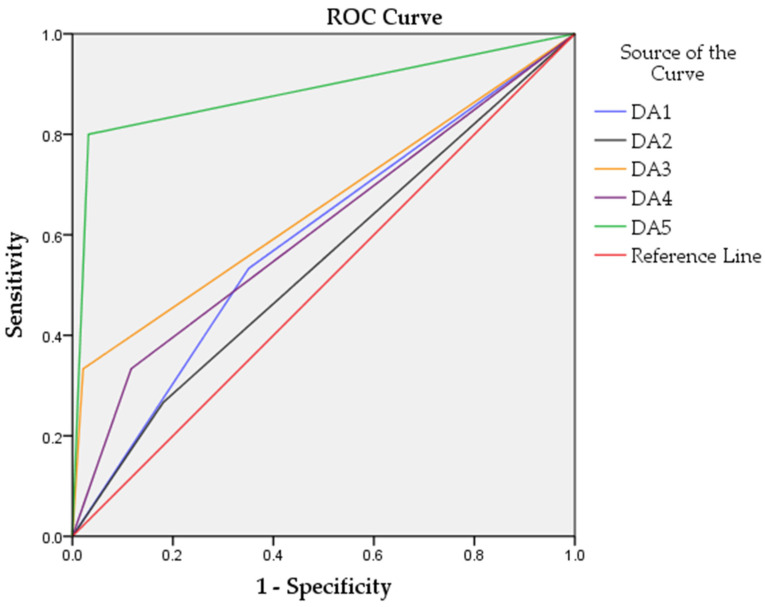
Performance of the discriminant function analyses models with different sets of biomarkers. ROC curves are represented in different colors for each model.

The DA5 model yielded the best results (Figure 3) and classified healthy and tumor samples with 95.4% accuracy, 97.9% sensitivity, 80% specificity, and receiver operating characteristic area under the curve (ROC AUC) of 0.884.

## 4. Discussion

This study presents a liquid biopsy-based multi-analyte classification model for tumor samples and healthy controls. The *AR* p.H875Y mutation plays a key role in this model. Androgen receptor alterations have been identified as some of the main drivers of castration-resistant prostate cancer [25]. The *AR* p.H875Y mutation has been predominantly found in prostate cancer [26], but this mutation has also been reported for breast cancer [27] and CRC [28]. However, to our knowledge, this is the first time that *AR* p.H875Y mutation has been reported for bladder, lung, stomach, ovarian, brain, and pancreas cancer. *AR* mutations have been predominantly studied in connection to prostate and breast cancer, especially treatment response [29,30]. We analyzed all predefined targets in all samples, not only the genes reported to be relevant in the specific cancer type. Considering this, we speculate that there is no literature concerning other tumors until now because other studies that analyzed this specific *AR* mutation focused primarily on breast and prostate cancer. Besides, we used a qPCR-based method to detect cfDNA mutations, which is shown to have a better sensitivity to detect low allele fraction variants than sequencing [31]. Still, the underlying mechanisms of the involvement of *AR* p.H875Y mutation in the carcinogenesis of these cancer types should be investigated. Nevertheless, our results suggest that *AR* p.H875Y mutation could be a promising biomarker for discriminating healthy subjects from cancer patients, especially CRC, bladder, and prostate. 

Here, we describe a model consisting of two steps, sorting samples in two groups—with and without an *AR* mutation (AR+ and AR– respectively) and consequently classifying the AR– group in cancer patients and healthy subjects (95.4% accuracy, 97.9% sensitivity, 80% specificity, 0.884 ROC AUC). The classification models, based solely on mutations, cfDNA methylation, or miRNAs, showed poor specificity (DA2 26.7%, DA3 33.3%, and DA4 33.3%, respectively). Combining all the analyzed biomarkers improved the specificity to some extent (DA1 57.3%); however, the sensitivity declined. The large number of biomarkers included in the DA1 model decreases the classifier’s performance since some contain redundant and superficial information. To alleviate the effects of this so-called “curse of dimensionality,” also known as the “Hughes phenomenon” [32,33], the number of biomarkers included in the model should be decreased. Hence, biomarkers selection was carried out, and a classification model was performed based on the most relevant biomarkers (DA5), displaying the best results (Table 3, Figure 3). 

Interestingly, our results demonstrate that the combination of three different analytes could improve the performance of a classification model. Each analyte type provides distinct information and adds value to the classification model, highlighting the importance of a multi-analyte-based liquid biopsy test for cancer detection [3,34]. 

Although cfDNA concentration has been previously suggested as a biomarker for cancer detection [35], our results did not support these findings. The healthy subjects in this study exhibited a higher amount of cfDNA plasma concentrations than patients with stage I tumors which has been already reported [36]. Plasma cfDNA present in healthy subjects is not unusual; however, the main contributor of cfDNA is the apoptosis of hematopoietic cells [37]. The cfDNA profile of a cancer patient differs from a healthy individual, whereby it consists of fragments originating from tumor cells, also called circulating tumor DNA (ctDNA). Additionally, these DNA fragments have a specific footprint indicating the tissue of origin [38]. Although we did not estimate the percentage of ctDNA of the total plasma cfDNA, we detected a higher mutational burden in cancer patients compared to healthy individuals. 

Nonetheless, some genomic aberrations were detected among the control group (Supplementary Table S3). Somatic mutations in healthy tissues have been previously reported [39]. Since no follow-up of the participants was conducted, we cannot know if the healthy subjects harboring these somatic mutations developed cancer. However, these subjects were declared cancer-free at sample collection. 

Our results suggest that plasma cfDNA could serve rather as a monitor for disease progression since cfDNA concentration correlated with cancer stage regardless of cancer type. However, mutational burden and miRNA expression and cfDNA methylation should be taken into account [40]. 

As mentioned earlier, one key limitation of this study is the lack of follow-up in addition to the small number of healthy controls. Despite the small size of the control group, it sufficed to observe several statistically significant results since it was matched to reflect the average sample size of each cancer group. Another potential limitation is the clinical utility of this model, as the three different analytes require separate sample processing and analysis. Nevertheless, a blood-based test is a minimally invasive procedure in contrast to a tissue biopsy and is more frequently accepted by patients than other screening procedures such as colonoscopy [41] or a fecal immunochemical test (FIT) [6] in the case of CRC. Thus, the screening methods themselves directly affect compliance and should be therefore optimized.

Liquid biopsy markers such as cfDNA mutations, cfDNA methylation, and CTCs are already successfully applied as prognostic and predictive tools for treatment response in several tumor types and monitoring tools for disease progression [42,43,44,45], as well as for disease screening [5]. Yet, there are still no clinically approved tests for a broader cancer screening of the population. Despite the limitations of this study, our results indicate that pan-cancer detection could be achieved through the combination of genetic and epigenetic biomarkers in plasma.

## 5. Conclusions

In this study, we created a liquid biopsy-based classification model allowing the discrimination between healthy controls and patients with various solid tumors. We demonstrated that combining several analytes improves the performance of the test. Nevertheless, a bigger prospective cohort is required to confirm the clinical utility of this classification model and assess whether a subclassification of the different cancer types is possible.

## Figures and Tables

**Table 1:** Patients characteristics.

Subjects	*n*	Age Mean (95% CI)	Sex *n* (%)		Cancer stage *n* (%)			Family History of Cancer *n* (%)	BMI Mean	Current Infection *n* (%)
			Female	Male	I	II	III			
Healthy	15	54.60 (46.23–62.97) ^b,c,e,f^	8 (53)	7 (47)	0 (0)	0 (0)	0 (0)	0 (0)	25.47	0(0)
Bladder	20	70.75 (66.05–75.45) ^a^	3 (15)	17 (85)	9 (45)	6 (30)	5 (25)	0 (0)	26.26	0 (0)
Brain	9	63.22 (54.33–72.12)	3 (33)	6 (67)	2 (22)	4 (44)	3 (33)	0 (0)	25.22	0 (0)
Breast	30	63.67 (59.39–67.94)	29 (97)	1 (3)	8 (27)	16 (53)	6 (20)	0 (0)	25.5	0 (0)
Colorectal	28	66.57 (62.81–70.34) ^a^	14 (50)	14 (50)	3 (11)	19 (68)	6 (21)	0 (0)	24.65 ^d^	0 (0)
Lung	29	62.62 (59.05–66.19)	4 (14)	25 (86)	1 (3)	12 (41)	16 (55)	5 (17)	25.77	1 (3)
Ovarian	19	60.95 (56.44–65.46)	19 (100)	0 (0)	0 (0)	4 (21)	15 (79)	0 (0)	28.19 ^c^	0 (0)
Prostate	27	66.70 (63.15–70.26) ^a^	0 (0)	27 (100)	16 (59)	7 (26)	4 (15)	1 (4)	26.08	2 (7)
Stomach	23	69.35 (65.04–73.65) ^a^	15 (65)	8 (35)	0 (0)	8 (35)	15 (65)	0 (0)	22.98	0 (0)
Pancreas	12	67.08 (61.77–72.40)	6 (50)	6 (50)	0 (0)	5 (42)	7 (58)	0 (0)	24.41	0 (0)
Total	212	64.55 (63.08–66.01)	101 (48)	111 (52)	39 (18)	81 (38)	77 (36)	6 (3)	25.45	3 (1)

Lower case letters indicate the group with a significantly different value at *p* < 0.05: ^a^ healthy, ^b^ bladder, ^c^ CRC, ^d^ ovarian, ^e^ prostate, ^f^ pancreas.

**Table 2:** Biomarkers with the highest correlations for each cancer type.

Cancer Type	cfDNA Mutations	cfDNA Methylation	miRNAs
Bladder	*AR* (COSM238555), *TP53* (COSM10758)	-	miR-17-5p
Brain	-	*MLH1* m%, *GATA5* m%	miR-133a-3p
Breast	*AR* (COSM238555), *TP53* (COSM10758)	*MDR1* m%	miR-17-5p
CRC	*AR* (COSM238555), *TP53* (COSM10758)	-	miR-17-5p,
Lung	*TP53* (COSM10758)	-	miR-17-5p, miR-92a-3p, miR-155-5p
Ovarian	-	-	miR-29c-3p, miR-92a-3p, miR-101-3p, miR-148b-3p
Pancreas	-	*SFN* m%	miR-27a-3p, miR-29c-3p, miR-148b-3p
Prostate	*AR* (COSM238555)	-	miR-17-5p, miR-26a-5p
Stomach	*APC* (COSM18561)	-	miR-20a-5p, miR-21-5p

**Table 3:** Discriminant analysis for classification of AR– samples in healthy and tumor samples.

Discriminant Analysis Model	Accuracy %	Sensitivity %	Specificity %	ROC AUC	*n* of Biomarkers
DA1	55.9	46.7	57.3	0.591	119
DA2	73.9	81.3	26.7	0.543	64
DA3	89.2	97.9	33.3	0.656	12
DA4	80.2	87.5	33.3	0.608	43
DA5	95.4	97.9	80.0	0.884	18

## Data Availability

The data generated in this study are available upon reasonable request from the corresponding author.

## Supplementary Materials

The following are available online at www.mdpi.com/article/10.3390/cancers14020462/s1, Table S1: Assays for the mutation analysis of cell-free DNA, Table S2: Primer sequences used for methylation analysis of cell-free DNA, Table S3: Assays used for miRNA analysis, Table S4: Mutation frequencies in all samples, Figure S1: Correlation matrix of all variables, Figure S2: Correlation plots for each cancer type, Figure S3: Heatmap of the cell-free DNA methylation, Figure S4: Heatmap of the miRNAs levels.
