# Supplementary material for: Comprehensive Approach to Distinguish Patients with Solid Tumors from Healthy Controls by Combining Androgen Receptor Mutation p.H875Y with Cell-Free DNA Methylation and Circulating miRNAs
Source: Cancers (Basel). 2022 Jan 17;14(2):462. doi: 10.3390/cancers14020462 (PMC8774173; doi:10.3390/cancers14020462)
Supplement: Supplementary file 1 [file cancers-14-00462-s001.zip › cancers-1542395-supplementary/Supplementary Materials revised cancers-1542395 vs110122.pdf]

cancers

MDPI

Comprehensive approach to distinguish patients with solid tumors from healthy controls by combining androgen receptor mutation p.H875Y with cell-free DNA methylation and circulating miRNAs Elena Tomeva, Olivier J. Switzeny , Clemens Heitzinger, Berit Hippe, and Alexander G. Haslberger

This file contains supplementary materials for the manuscript Comprehensive approach to distinguish patients with solid tumors from healthy controls by combining androgen receptor mutation p.H875Y with cell-free DNA methylation and circulating miRNAs by Tomeva E., Switzeny OJ., Heitzinger C., Hippe B., and Haslberger AG (2021)

List of tables

Supplementary Table S1. Assays for the mutation analysis of cell-free DNA. 1
Supplementary Table S2. Primer sequences used for methylation analysis of cell-free DNA. 4
Supplementary Table S3. Assays used for miRNA analysis.. 5
Supplementary Table S4. Mutation frequencies in all samples... 7

List of figures

Supplementary Figure S1. Correlation matrix of all variables. 10
Supplementary Figure S2. Correlation plots for each cancer type. 11
Supplementary Figure S3. Heatmap of the cell-free DNA methylation....................................... 12
Supplementary Figure S4. Heatmap of the miRNAs levels. 13

Supplementary Table S1. Assays for the mutation analysis of cell-free DNA.
ΔCt values for each sample were calculated as followed: ΔCt=Ct(Mu Assay)-Ct(Rf Assay). If a ΔCt value for a sample was beneath the cut-off ΔCt value a presence of a mutation was concluded. Ct threshold cycle, mu mutation, rf reference, AA amino acid

Gene name	Cosmic ID	Target Information	AA mutation type	Cut-off ΔCt value for the presence of a mutation	TaqMan™ Mutation Detection Assays ID
AKT1	COSM33765	mu	Substitution - Missense	<8.0	Hs00000986_mu
	-	rf			Hs00001010_rf
APC	COSM13127	mu	Substitution - Nonsense	<6.5	Hs00000945_mu
	COSM18561	mu	Insertion - Frameshift	<5.0	Hs00000958_mu
	-	rf			Hs00001012_rf
AR	COSM236693	mu	Substitution - Missense	<9.0	custom assay
	COSM238553	mu	Substitution - Missense	<9.0	custom assay
	COSM238555	mu	Substitution - Missense	<9.0	custom assay
	-	rf			custom assay

BRAF	COSM476	mu	Substitution - Missense	<7.0	custom assay
	-	rf			Hs00000172_rf
CTNNB1	COSM5661	mu	Substitution - Missense	<9.0	Hs00000863_mu
	COSM5662	mu	Substitution - Missense	<9.0	Hs00000864_mu
	COSM5663	mu	Substitution - Missense	<9.0	Hs00000865_mu
	COSM5664	mu	Substitution - Missense	<9.0	Hs00000866_mu
	COSM5677	mu	Substitution - Missense	<9.0	Hs00000875_mu
	-	rf			Hs00001014_rf
EGFR	COSM6224	mu	Substitution - Missense	<9.0	Hs00000102_mu
	COSM6240	mu	Substitution - Missense	<9.0	Hs00000106_mu
	-	rf			Hs00000173_rf
ERBB2	COSM12558	mu	Insertion - In frame	<8.5	Hs00001463_mu
	COSM14060	mu	Substitution - Missense	<7.7	Hs00001565_mu
	COSM14062	mu	Substitution - Missense	<6.8	Hs00001142_mu
	COSM48358	mu	Substitution - Missense	<9.0	custom assay
	-	rf			Hs00001366_rf
ESR1	COSM4745827	mu	Substitution - Missense	<9.0	custom assay
	-	rf			custom assay
FBXW7	COSM22932	mu	Substitution - Missense	<4.2	Hs00001410_mu
	COSM22965	mu	Substitution - Missense	<4.2	Hs00001070_mu
	-	rf			Hs00001368_rf
FGFR3	COSM715	mu	Substitution - Missense	<5.2	Hs00000812_mu
	COSM718	mu	Substitution - Missense	<8.5	Hs00000814_mu
	-	rf			Hs00001015_rf
GNAS	COSM27887	mu	Substitution - Missense	<9.0	Hs00000976_mu
	-	rf			Hs00001017_rf
HRAS	COSM499	mu	Substitution - Missense	<8.4	Hs00000787_mu
	-	rf			Hs00001018_rf
IDH1	COSM28746	mu	Substitution - Missense	<8.0	Hs00000981_mu
	COSM28747	mu	Substitution - Missense	<8.2	Hs00000982_mu
	-	rf			Hs00001019_rf
KRAS	COSM516	mu	Substitution - Missense	<9.0	Hs00000113_mu
	COSM517	mu	Substitution - Missense	<8.0	Hs00000115_mu
	COSM518	mu	Substitution - Missense	<8.0	Hs00000117_mu

	COSM520	mu	Substitution - Missense	<5.6	Hs00000119_mu
	COSM521	mu	Substitution - Missense	<4.0	Hs00000121_mu
	COSM522	mu	Substitution - Missense	<5.7	Hs00000123_mu
	COSM532	mu	Substitution - Missense	<5.8	Hs00000131_mu
	-	rf			Hs00000174_rf
MED12	COSM131596	mu	Substitution - Missense	<7.0	custom assay
	-	rf			custom assay
NRAS	COSM580	mu	Substitution - Missense	<8.0	Hs00000804_mu
	COSM584	mu	Substitution - Missense	<8.0	Hs00000808_mu
	-	rf			Hs00001023_rf
PIK3CA	COSM760	mu	Substitution - Missense	<4.0	Hs00000822_mu
	COSM763	mu	Substitution - Missense	<5.5	Hs00000824_mu
	COSM775	mu	Substitution - Missense	<8.3	Hs00000831_mu
	COSM776	mu	Substitution - Missense	<8.0	Hs00000832_mu
	-	rf			Hs00001025_rf
SMAD4	COSM14122	mu	Substitution - Missense	<6.0	Hs00001433_mu
	-	rf			Hs00001380_rf
TERT	COSM1716558	mu	Intron variant	<8.0	custom assay
	COSM1716559	mu	Intron variant	<8.0	custom assay
	-	rf			custom assay
TP53	COSM10645	mu	Substitution - Missense	<9.0	Hs00000887_mu
	COSM10648	mu	Substitution - Missense	<8.8	Hs00000888_mu
	COSM10654	mu	Substitution - Nonsense	<8.2	Hs00001003_mu
	COSM10656	mu	Substitution - Missense	<5.9	Hs00000889_mu
	COSM10659	mu	Substitution - Missense	<7.6	Hs00000890_mu
	COSM10660	mu	Substitution - Missense	<6.6	Hs00001004_mu
	COSM10662	mu	Substitution - Missense	<8.0	Hs00001005_mu
	COSM10663	mu	Substitution - Nonsense	<7.0	Hs00000891_mu
	COSM10670	mu	Substitution - Missense	<8.3	Hs00000893_mu
	COSM10690	mu	Substitution - Missense	<7.1	Hs00000894_mu
	COSM10704	mu	Substitution - Missense	<8.0	Hs00000895_mu
	COSM10705	mu	Substitution - Nonsense	<7.8	Hs00000896_mu
	COSM10722	mu	Substitution - Missense	<6.6	Hs00000901_mu

	COSM10733	mu	Substitution - Nonsense	<8.6	Hs00000903_mu
	COSM10742	mu	Substitution - Missense	<7.4	Hs00000905_mu
	COSM10758	mu	Substitution - Missense	<8.3	Hs00000906_mu
	COSM10790	mu	Substitution - Missense	<7.0	Hs00000909_mu
	COSM10808	mu	Substitution - Missense	<9.0	Hs00000910_mu
	COSM10817	mu	Substitution - Missense	<8.1	Hs00000914_mu
	COSM10863	mu	Substitution - Missense	<7.0	Hs00000915_mu
	COSM10889	mu	Substitution - Missense	<3.0	Hs00001006_mu
	COSM10891	mu	Substitution - Missense	<8.0	Hs00000917_mu
	COSM11073	mu	Substitution - Nonsense	<8.0	Hs00000921_mu
	COSM6549	mu	Substitution - Missense	<9.0	Hs00000885_mu
	COSM6932	mu	Substitution - Missense	<6.6	Hs00000886_mu
	-	rf			Hs00001027_rf
VHL	COSM14305	mu	Substitution - Missense	<5.1	Hs00001107_mu
	COSM14372	mu	Substitution - Nonsense	<7.8	Hs00000949_mu
	COSM17612	mu	Substitution - Nonsense	<7.5	Hs00001113_mu
	-	rf			Hs00001028_rf

Supplementary Table S2. Primer sequences used for methylation analysis of cell-free DNA. fwd forward primer, rev reverse primer, bp base pairs

Gene	Primer	Sequence '5->3'	Amplicon length bp
SEPT9	Fwd	CTGCCCACCAGCCATCAT	62
	Rev	CCGAAATGATCCCATCCAGC	
MLH1	Fwd	TATTCGTGCTCAGCCTCGTA	119
	Rev	CGTTATTTGGTGGTGGAGCC	
MGMT	Fwd	CGCCCCTAGAACGCTTTG	74
	Rev	GACACTCACCAAGTCGCAAA	
GATA5	Fwd	AGAAGGGGCGGGCG	100
	Rev	AAGACTGGAAGCCCGGG	
GSTP1	Fwd	CTCCGGGGACTCCAGGG	100
	Rev	CGCTCTTCTGGAGGGTCC	
SFN	Fwd	CCGAACGCTATGAGGACATG	114
	Rev	CGCCCACCACGTTCTTATAG	
MDR1	Fwd	GGTGGGAGGAAGCATCGT	100
	Rev	GGCAGAGTTGGGGGTCT	
VIM	Fwd	GCTTCTCGCTAGGTCCCTAT	100
	Rev	CCGAGGGCGCTGTTTTTATA	

SHOX2	Fwd	TTAAACATCCGAGCTGCTGG	115
	Rev	CGACCCTAAACGCTTAACCC	
ALKBH3	Fwd	CTACCCGGACTGAGGACTG	100
	Rev	CTCCAGCAACTCCCAATCAC	
APC	Fwd	CTAGGGCTAGGCAGGCTG	100
	Rev	ATCCAGCGGATTACACAGCT	
RASSF1A	Fwd	GAGCTGGCACCCGCT	100
	Rev	GCCGTGTGGGGTTGC	

Supplementary Table S3. Assays used for miRNA analysis.

TaqMan™ Advanced miRNA Assay ID	miRNA
478575_mir	hsa-let-7a-5p
477863_mir	hsa-miR-101-3p
478225_mir	hsa-miR-106a-5p
477887_mir	hsa-miR-126-3p
478511_mir	hsa-miR-133a-3p
478501_mir	hsa-miR-141-3p
477911_mir	hsa-miR-142-5p
477912_mir	hsa-miR-143-3p
477916_mir	hsa-miR-145-5p
477814_mir	hsa-miR-148a-3p
477824_mir	hsa-miR-148b-3p
477927_mir	hsa-miR-155-5p
478447_mir	hsa-miR-17-5p
477935_mir	hsa-miR-182-5p
477940_mir	hsa-miR-186-5p
477957_mir	hsa-miR-195-5p
477967_mir	hsa-miR-205-5p
478586_mir	hsa-miR-20a-5p
478293_mir	cel-miR-39-3p
477975_mir	hsa-miR-21-5p
477970_mir	hsa-miR-210-3p
477985_mir	hsa-miR-22-3p
477987_mir	hsa-miR-22-5p
477981_mir	hsa-miR-221-3p
477982_mir	hsa-miR-222-3p
477983_mir	hsa-miR-223-3p
477986_mir	hsa-miR-224-5p
478532_mir	hsa-miR-23a-3p
477994_mir	hsa-miR-25-3p
477995_mir	hsa-miR-26a-5p
478384_mir	hsa-miR-27a-3p
479229_mir	hsa-miR-29c-3p

479448_mir	hsa-miR-30a-5p
478015_mir	hsa-miR-31-5p
478048_mir	hsa-miR-34a-5p
478074_mir	hsa-miR-375-3p
478107_mir	hsa-miR-451a
478138_mir	hsa-miR-497-5p
477827_mir	hsa-miR-92a-3p
477879_mir	hsa-miR-124-3p
477977_mir	hsa-miR-218-5p
477937_mir	hsa-miR-183-5p
478316_mir	hsa-miR-203a-3p
477875_mir	hsa-miR-1225-3p
478215_mir	hsa-miR-96-5p
478214_mir	hsa-miR-9-5p
477860_mir	hsa-miR-16-5p
478418_mir	hsa-miR-26b-5p

Supplementary Table S4. Mutation frequencies in all samples.
The frequencies of the detected mutations are depicted as an absolute value and percentage of the respective study group (in round brackets).

		Healthy	Bladder	Brain	Breast	CRC	Lung	Ovarian	Prostate	Stomach	Pancreas	All
	N	15	20	9	30	28	29	19	27	23	12	212
	Females	8	3	3	29	14	4	19	0	15	6	101
	Male	7	17	6	1	14	25	0	27	8	6	111
AKT1	COSM33765	0 (0 %)	0 (0 %)	0 (0 %)	2 (7 %)	1 (4 %)	1 (3 %)	0 (0 %)	1 (4 %)	0 (0 %)	0 (0 %)	5 (2 %)
APC	COSM13127	0 (0 %)	0 (0 %)	0 (0 %)	4 (13 %)	3 (11 %)	2 (7 %)	1 (5 %)	2 (7 %)	1 (4 %)	3 (25 %)	16 (8 %)
APC	COSM18561	0 (0 %)	4 (20 %)	2 (22 %)	7 (23 %)	5 (18 %)	6 (21 %)	5 (26 %)	5 (19 %)	7 (30 %)	4 (33 %)	45 (21 %)
AR	COSM238555	0 (0 %)	16 (80 %)	1 (11 %)	18 (60 %)	24 (86 %)	14 (48 %)	3 (16 %)	18 (67 %)	6 (26 %)	1 (8 %)	101 (48 %)
AR	COSM236693	0 (0 %)	3 (15 %)	1 (11 %)	6 (20 %)	4 (14 %)	2 (7 %)	2 (11 %)	6 (22 %)	2 (9 %)	0 (0 %)	26 (12 %)
AR	COSM238553	0 (0 %)	0 (0 %)	0 (0 %)	2 (7 %)	1 (4 %)	2 (7 %)	0 (0 %)	1 (4 %)	0 (0 %)	0 (0 %)	6 (3 %)
BRAF	COSM476	0 (0 %)	3 (15 %)	2 (22 %)	3 (10 %)	4 (14 %)	4 (14 %)	2 (11 %)	1 (4 %)	0 (0 %)	2 (17 %)	21 (10 %)
CTNNB1	COSM5663	2 (13 %)	4 (20 %)	1 (11 %)	2 (7 %)	4 (14 %)	1 (3 %)	0 (0 %)	0 (0 %)	1 (4 %)	0 (0 %)	15 (7 %)
CTNNB1	COSM5664	0 (0 %)	2 (10 %)	0 (0 %)	1 (3 %)	0 (0 %)	0 (0 %)	0 (0 %)	1 (4 %)	0 (0 %)	0 (0 %)	4 (2 %)
CTNNB1	COSM5662	0 (0 %)	0 (0 %)	0 (0 %)	0 (0 %)	0 (0 %)	0 (0 %)	2 (11 %)	1 (4 %)	3 (13 %)	1 (8 %)	7 (3 %)
CTNNB1	COSM5661	0 (0 %)	3 (15 %)	2 (22 %)	2 (7 %)	3 (11 %)	0 (0 %)	0 (0 %)	0 (0 %)	1 (4 %)	0 (0 %)	11 (5 %)
CTNNB1	COSM5677	0 (0 %)	0 (0 %)	0 (0 %)	0 (0 %)	0 (0 %)	0 (0 %)	0 (0 %)	0 (0 %)	0 (0 %)	0 (0 %)	0 (0 %)
EGFR	COSM6224	8 (53 %)	9 (45 %)	1 (11 %)	8 (27 %)	17 (61 %)	13 (45 %)	9 (47 %)	12 (44 %)	11 (48 %)	9 (75 %)	97 (46 %)
EGFR	COSM6223	0 (0 %)	0 (0 %)	0 (0 %)	0 (0 %)	0 (0 %)	0 (0 %)	0 (0 %)	0 (0 %)	0 (0 %)	0 (0 %)	0 (0 %)
EGFR	COSM6240	0 (0 %)	1 (5 %)	0 (0 %)	1 (3 %)	1 (4 %)	1 (3 %)	0 (0 %)	2 (7 %)	1 (4 %)	0 (0 %)	7 (3 %)
ERBB2	COSM48358	0 (0 %)	0 (0 %)	1 (11 %)	0 (0 %)	0 (0 %)	1 (3 %)	0 (0 %)	0 (0 %)	1 (4 %)	0 (0 %)	3 (1 %)
ERBB2	COSM14060	0 (0 %)	1 (5 %)	1 (11 %)	1 (3 %)	1 (4 %)	2 (7 %)	1 (5 %)	2 (7 %)	2 (9 %)	0 (0 %)	11 (5 %)
ERBB2	COSM12558	0 (0 %)	0 (0 %)	0 (0 %)	1 (3 %)	0 (0 %)	0 (0 %)	0 (0 %)	0 (0 %)	1 (4 %)	0 (0 %)	2 (1 %)
ERBB2	COSM14062	0 (0 %)	3 (15 %)	2 (22 %)	2 (7 %)	7 (25 %)	4 (14 %)	1 (5 %)	1 (4 %)	2 (9 %)	0 (0 %)	22 (10 %)
ESR1	COSM4745827	0 (0 %)	0 (0 %)	0 (0 %)	1 (3 %)	2 (7 %)	4 (14 %)	0 (0 %)	1 (4 %)	0 (0 %)	0 (0 %)	8 (4 %)
FBXW7	COSM22965	0 (0 %)	1 (5 %)	1 (11 %)	1 (3 %)	0 (0 %)	0 (0 %)	0 (0 %)	0 (0 %)	0 (0 %)	0 (0 %)	3 (1 %)
FBXW7	COSM22932	0 (0 %)	0 (0 %)	0 (0 %)	0 (0 %)	0 (0 %)	0 (0 %)	0 (0 %)	0 (0 %)	0 (0 %)	0 (0 %)	0 (0 %)
FGFR3	COSM715	0 (0 %)	0 (0 %)	0 (0 %)	1 (3 %)	0 (0 %)	1 (3 %)	0 (0 %)	0 (0 %)	0 (0 %)	0 (0 %)	2 (1 %)
FGFR3	COSM718	3 (20 %)	4 (20 %)	3 (33 %)	8 (27 %)	10 (36 %)	8 (28 %)	8 (42 %)	10 (37 %)	9 (39 %)	1 (8 %)	64 (30 %)
GNAS	COSM27887	0 (0 %)	0 (0 %)	0 (0 %)	2 (7 %)	1 (4 %)	1 (3 %)	0 (0 %)	1 (4 %)	0 (0 %)	0 (0 %)	5 (2 %)
HRAS	COSM483	0 (0 %)	0 (0 %)	0 (0 %)	0 (0 %)	0 (0 %)	0 (0 %)	0 (0 %)	0 (0 %)	0 (0 %)	0 (0 %)	0 (0 %)
HRAS	COSM499	0 (0 %)	0 (0 %)	0 (0 %)	0 (0 %)	0 (0 %)	0 (0 %)	0 (0 %)	0 (0 %)	0 (0 %)	0 (0 %)	0 (0 %)
IDH1	COSM28746	0 (0 %)	1 (5 %)	0 (0 %)	2 (7 %)	0 (0 %)	3 (10 %)	0 (0 %)	1 (4 %)	0 (0 %)	1 (8 %)	8 (4 %)
IDH1	COSM28747	0 (0 %)	1 (5 %)	0 (0 %)	2 (7 %)	0 (0 %)	3 (10 %)	0 (0 %)	1 (4 %)	0 (0 %)	0 (0 %)	7 (3 %)
KRAS	COSM521	0 (0 %)	3 (15 %)	0 (0 %)	0 (0 %)	0 (0 %)	1 (3 %)	0 (0 %)	3 (11 %)	0 (0 %)	1 (8 %)	8 (4 %)
KRAS	COSM520	0 (0 %)	0 (0 %)	1 (11 %)	0 (0 %)	0 (0 %)	0 (0 %)	0 (0 %)	0 (0 %)	0 (0 %)	0 (0 %)	1 (0 %)
KRAS	COSM516	0 (0 %)	1 (5 %)	0 (0 %)	0 (0 %)	1 (4 %)	0 (0 %)	1 (5 %)	1 (4 %)	0 (0 %)	0 (0 %)	4 (2 %)

KRAS	COSM532	0 (0 %)	1 (5 %)	1 (11 %)	2 (7 %)	2 (7 %)	2 (7 %)	1 (5 %)	3 (11 %)	1 (4 %)	2 (17 %)	15 (7 %)
KRAS	COSM522	0 (0 %)	0 (0 %)	0 (0 %)	1 (3 %)	0 (0 %)	0 (0 %)	1 (5 %)	1 (4 %)	0 (0 %)	0 (0 %)	3 (1 %)
KRAS	COSM518	0 (0 %)	0 (0 %)	0 (0 %)	0 (0 %)	0 (0 %)	0 (0 %)	0 (0 %)	0 (0 %)	0 (0 %)	0 (0 %)	0 (0 %)
KRAS	COSM517	2 (13 %)	1 (5 %)	1 (11 %)	1 (3 %)	0 (0 %)	1 (3 %)	0 (0 %)	1 (4 %)	0 (0 %)	0 (0 %)	7 (3 %)
MED12	COSM131596	0 (0 %)	3 (15 %)	0 (0 %)	3 (10 %)	3 (11 %)	4 (14 %)	1 (5 %)	6 (22 %)	0 (0 %)	0 (0 %)	20 (9 %)
NRAS	COSM584	0 (0 %)	2 (10 %)	1 (11 %)	1 (3 %)	2 (7 %)	5 (17 %)	1 (5 %)	1 (4 %)	4 (17 %)	1 (8 %)	18 (8 %)
NRAS	NRAS_580	0 (0 %)	1 (5 %)	0 (0 %)	0 (0 %)	2 (7 %)	0 (0 %)	0 (0 %)	0 (0 %)	0 (0 %)	0 (0 %)	3 (1 %)
PIK3CA	COSM763	0 (0 %)	6 (30 %)	0 (0 %)	4 (13 %)	5 (18 %)	4 (14 %)	4 (21 %)	5 (19 %)	2 (9 %)	3 (25 %)	33 (16 %)
PIK3CA	COSM775	0 (0 %)	1 (5 %)	2 (22 %)	4 (13 %)	2 (7 %)	4 (14 %)	1 (5 %)	5 (19 %)	3 (13 %)	0 (0 %)	22 (10 %)
PIK3CA	COSM776	0 (0 %)	1 (5 %)	0 (0 %)	0 (0 %)	0 (0 %)	1 (3 %)	2 (11 %)	2 (7 %)	0 (0 %)	1 (8 %)	7 (3 %)
PIK3CA	COSM760	1 (7 %)	5 (25 %)	1 (11 %)	2 (7 %)	1 (4 %)	1 (3 %)	1 (5 %)	0 (0 %)	3 (13 %)	1 (8 %)	16 (8 %)
SMAD4	COSM14122	0 (0 %)	0 (0 %)	0 (0 %)	0 (0 %)	0 (0 %)	1 (3 %)	1 (5 %)	1 (4 %)	2 (9 %)	1 (8 %)	6 (3 %)
TERT	COSM1716558	0 (0 %)	1 (5 %)	0 (0 %)	2 (7 %)	5 (18 %)	1 (3 %)	1 (5 %)	0 (0 %)	0 (0 %)	0 (0 %)	10 (5 %)
TERT	COSM1716559	0 (0 %)	6 (30 %)	2 (22 %)	5 (17 %)	11 (39 %)	7 (24 %)	3 (16 %)	6 (22 %)	4 (17 %)	2 (17 %)	46 (22 %)
TP53	COSM10648	0 (0 %)	3 (15 %)	0 (0 %)	6 (20 %)	8 (29 %)	3 (10 %)	1 (5 %)	2 (7 %)	2 (9 %)	4 (33 %)	29 (14 %)
TP53	COSM10659	0 (0 %)	2 (10 %)	0 (0 %)	3 (10 %)	3 (11 %)	3 (10 %)	0 (0 %)	2 (7 %)	2 (9 %)	2 (17 %)	17 (8 %)
TP53	COSM10662	1 (7 %)	2 (10 %)	0 (0 %)	4 (13 %)	3 (11 %)	3 (10 %)	2 (11 %)	3 (11 %)	1 (4 %)	4 (33 %)	23 (11 %)
TP53	COSM6549	1 (7 %)	0 (0 %)	1 (11 %)	4 (13 %)	6 (21 %)	6 (21 %)	5 (26 %)	4 (15 %)	1 (4 %)	4 (33 %)	32 (15 %)
TP53	COSM10656	0 (0 %)	1 (5 %)	0 (0 %)	3 (10 %)	1 (4 %)	0 (0 %)	1 (5 %)	0 (0 %)	0 (0 %)	0 (0 %)	7 (3 %)
TP53	COSM10779	0 (0 %)	0 (0 %)	0 (0 %)	0 (0 %)	0 (0 %)	0 (0 %)	0 (0 %)	0 (0 %)	0 (0 %)	0 (0 %)	0 (0 %)
TP53	COSM10660	0 (0 %)	2 (10 %)	0 (0 %)	3 (10 %)	4 (14 %)	3 (10 %)	0 (0 %)	1 (4 %)	1 (4 %)	1 (8 %)	15 (7 %)
TP53	COSM10654	0 (0 %)	3 (15 %)	0 (0 %)	4 (13 %)	6 (21 %)	6 (21 %)	2 (11 %)	5 (19 %)	2 (9 %)	3 (25 %)	31 (15 %)
TP53	COSM10704	0 (0 %)	0 (0 %)	0 (0 %)	5 (17 %)	4 (14 %)	1 (3 %)	1 (5 %)	2 (7 %)	1 (4 %)	3 (25 %)	17 (8 %)
TP53	COSM10758	0 (0 %)	12 (60 %)	0 (0 %)	12 (40 %)	16 (57 %)	15 (52 %)	5 (26 %)	8 (30 %)	6 (26 %)	3 (25 %)	77 (36 %)
TP53	COSM6932	0 (0 %)	0 (0 %)	0 (0 %)	2 (7 %)	0 (0 %)	2 (7 %)	1 (5 %)	1 (4 %)	2 (9 %)	2 (17 %)	10 (5 %)
TP53	COSM10705	0 (0 %)	3 (15 %)	0 (0 %)	8 (27 %)	6 (21 %)	3 (10 %)	2 (11 %)	2 (7 %)	2 (9 %)	4 (33 %)	30 (14 %)
TP53	COSM11073	0 (0 %)	0 (0 %)	0 (0 %)	1 (3 %)	1 (4 %)	3 (10 %)	1 (5 %)	0 (0 %)	1 (4 %)	1 (8 %)	8 (4 %)
TP53	COSM10663	0 (0 %)	1 (5 %)	1 (11 %)	5 (17 %)	1 (4 %)	2 (7 %)	1 (5 %)	1 (4 %)	0 (0 %)	2 (17 %)	14 (7 %)
TP53	COSM10808	0 (0 %)	0 (0 %)	0 (0 %)	2 (7 %)	1 (4 %)	0 (0 %)	2 (11 %)	0 (0 %)	0 (0 %)	1 (8 %)	6 (3 %)
TP53	COSM10889	0 (0 %)	2 (10 %)	0 (0 %)	4 (13 %)	2 (7 %)	1 (3 %)	1 (5 %)	1 (4 %)	2 (9 %)	5 (42 %)	18 (8 %)
TP53	COSM10891	0 (0 %)	1 (5 %)	0 (0 %)	5 (17 %)	2 (7 %)	4 (14 %)	1 (5 %)	1 (4 %)	3 (13 %)	1 (8 %)	18 (8 %)
TP53	COSM10690	1 (7 %)	3 (15 %)	0 (0 %)	7 (23 %)	5 (18 %)	3 (10 %)	1 (5 %)	4 (15 %)	5 (22 %)	5 (42 %)	34 (16 %)
TP53	COSM10742	0 (0 %)	3 (15 %)	0 (0 %)	8 (27 %)	5 (18 %)	5 (17 %)	0 (0 %)	2 (7 %)	3 (13 %)	2 (17 %)	28 (13 %)
TP53	COSM10645	0 (0 %)	0 (0 %)	0 (0 %)	2 (7 %)	1 (4 %)	1 (3 %)	0 (0 %)	0 (0 %)	0 (0 %)	1 (8 %)	5 (2 %)
TP53	COSM10670	0 (0 %)	0 (0 %)	0 (0 %)	1 (3 %)	0 (0 %)	0 (0 %)	0 (0 %)	0 (0 %)	0 (0 %)	0 (0 %)	1 (0 %)
TP53	COSM10722	0 (0 %)	2 (10 %)	0 (0 %)	3 (10 %)	2 (7 %)	0 (0 %)	0 (0 %)	1 (4 %)	0 (0 %)	1 (8 %)	9 (4 %)
TP53	COSM10733	0 (0 %)	2 (10 %)	0 (0 %)	6 (20 %)	1 (4 %)	2 (7 %)	2 (11 %)	3 (11 %)	3 (13 %)	0 (0 %)	19 (9 %)
TP53	COSM10817	0 (0 %)	1 (5 %)	0 (0 %)	2 (7 %)	2 (7 %)	1 (3 %)	1 (5 %)	0 (0 %)	0 (0 %)	0 (0 %)	7 (3 %)
TP53	COSM10790	0 (0 %)	0 (0 %)	0 (0 %)	6 (20 %)	2 (7 %)	3 (10 %)	2 (11 %)	4 (15 %)	0 (0 %)	2 (17 %)	19 (9 %)

TP53	COSM10863	1 (7 %)	1 (5 %)	0 (0 %)	4 (13 %)	3 (11 %)	4 (14 %)	0 (0 %)	2 (7 %)	0 (0 %)	1 (8 %)	16 (8 %)
VHL	COSM14305	0 (0 %)	0 (0 %)	1 (11 %)	1 (3 %)	1 (4 %)	0 (0 %)	0 (0 %)	1 (4 %)	0 (0 %)	0 (0 %)	4 (2 %)
VHL	COSM14372	0 (0 %)	0 (0 %)	0 (0 %)	2 (7 %)	1 (4 %)	0 (0 %)	0 (0 %)	2 (7 %)	0 (0 %)	1 (8 %)	6 (3 %)
VHL	COSM17612	0 (0 %)	5 (25 %)	0 (0 %)	4 (13 %)	3 (11 %)	2 (7 %)	0 (0 %)	1 (4 %)	3 (13 %)	0 (0 %)	18 (8 %)

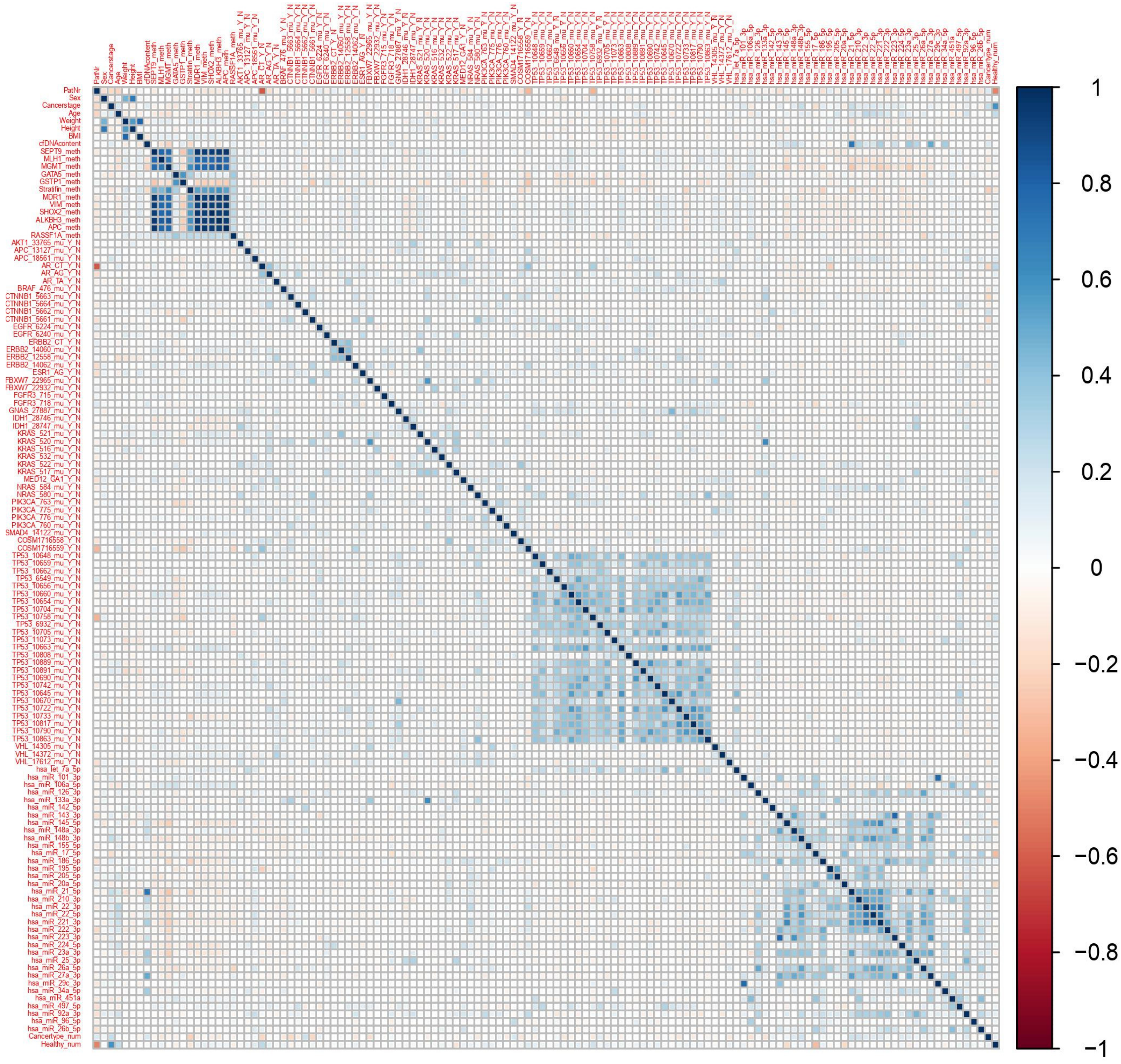

Supplementary Figure S1. Correlation matrix of all variables.
A correlation coefficient of ±1 indicates a perfect correlation between the two variables, while a coefficient of 0 indicates no correlation at all.

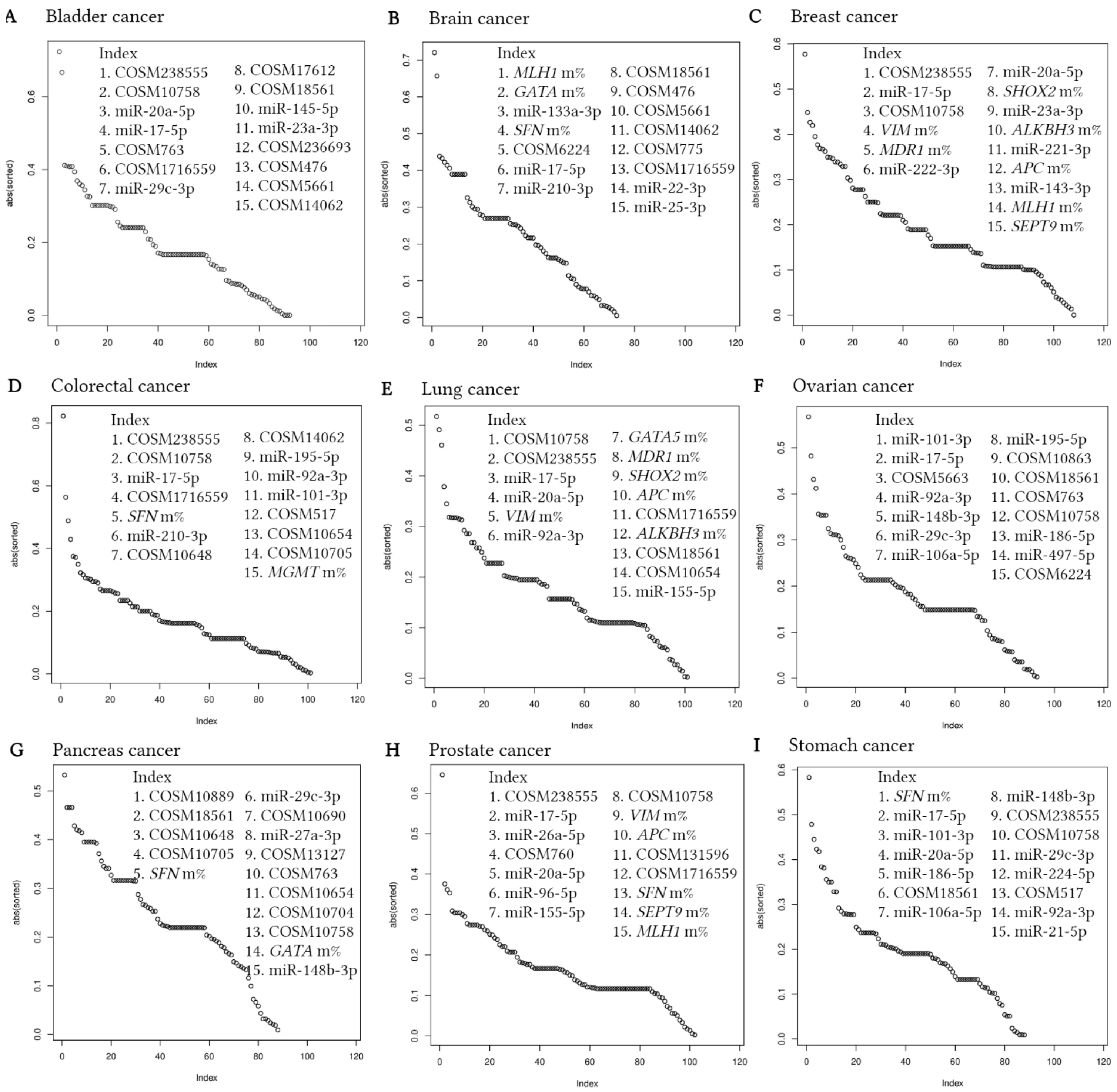

Supplementary Figure S2. Correlation plots for each cancer type.
The correlation coefficient of each biomarker is plotted on the y axis. An index value is assigned to each biomarker according to the value of its correlation coefficient (ranked from highest to lowest value) and plotted on the x axis. The 15 biomarkers with the highest correlation coefficients are displayed in the legends of the plots for each cancer type **A-I**

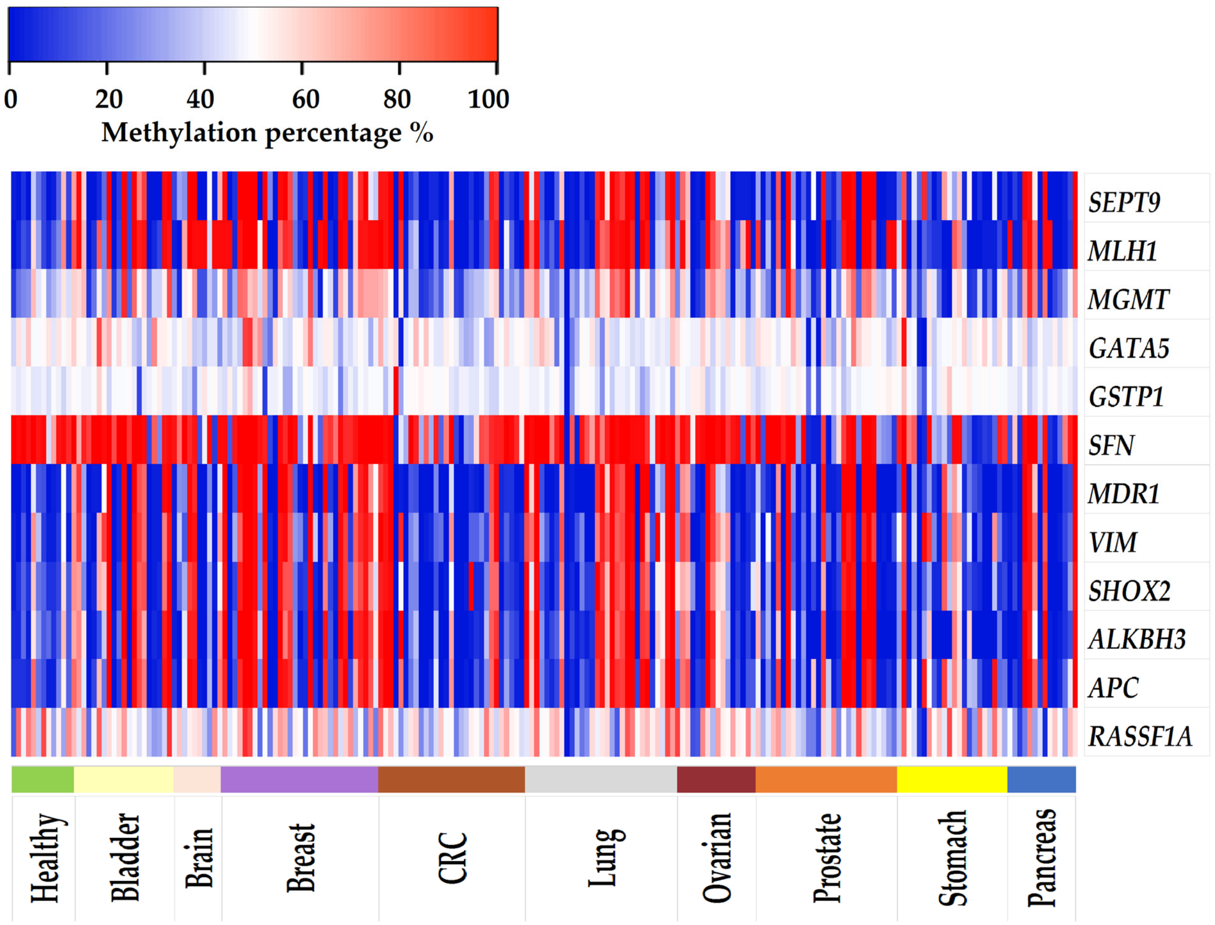

Supplementary Figure S3. Heatmap of the cell-free DNA methylation.
Columns represent the samples clustered by group, the rows represent the cell-free DNA methylation levels in red (high methylation) and in blue (low methylation).

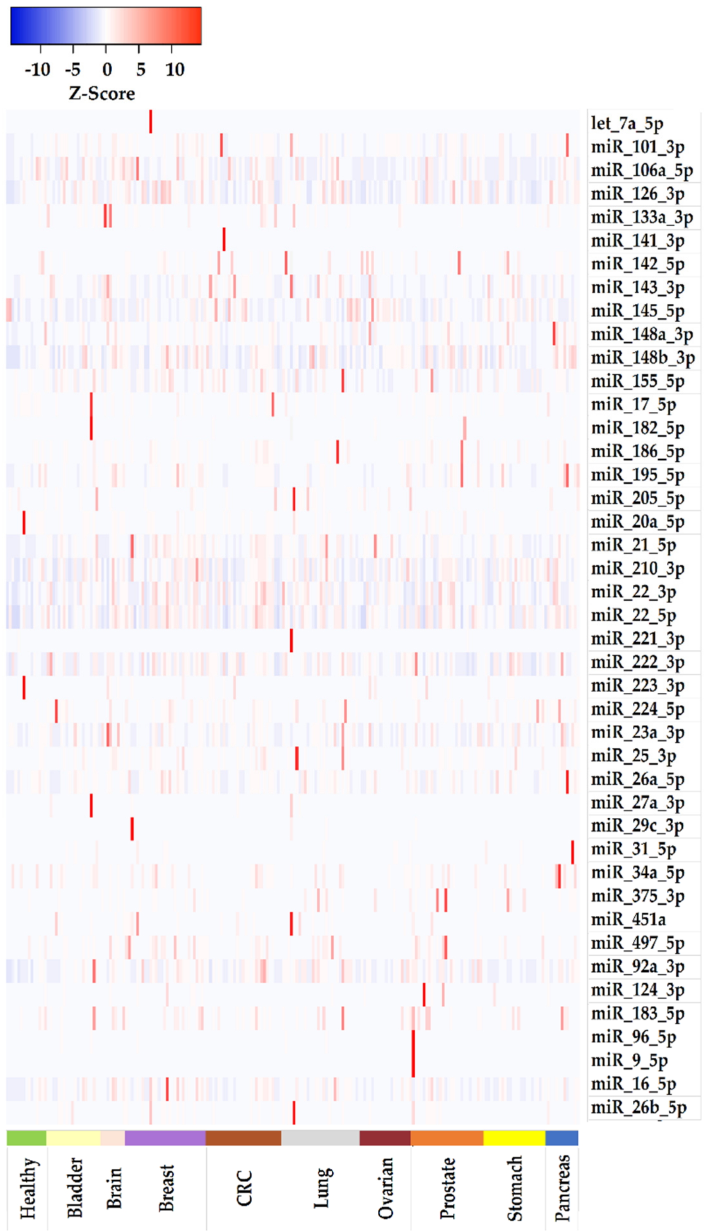

Supplementary Figure S4. Heatmap of the miRNAs levels.
Columns represent the samples clustered by group, the rows represent the miRNAs in red (up-regulated) and in blue (down-regulated).
