# Supplementary figures and images for: Comprehensive Approach to Distinguish Patients with Solid Tumors from Healthy Controls by Combining Androgen Receptor Mutation p.H875Y with Cell-Free DNA Methylation and Circulating miRNAs
Source: Cancers (Basel). 2022 Jan 17;14(2):462. doi: 10.3390/cancers14020462 (PMC8774173; doi:10.3390/cancers14020462)

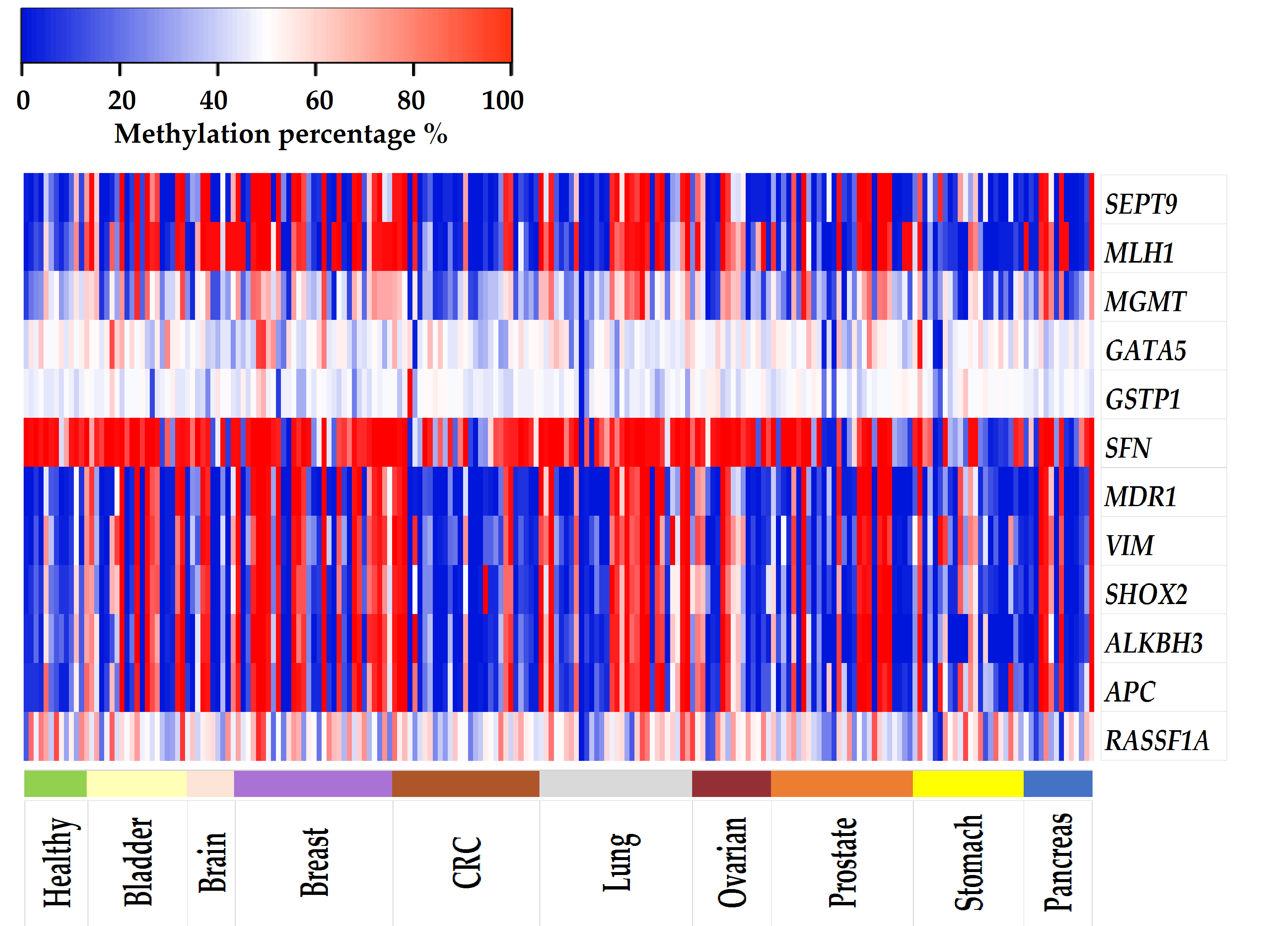

Supplement: Supplementary file 1 [file cancers-14-00462-s001.zip › cancers-1542395-supplementary/Supplementary Figure S3. Heatmap of the cell-free DNA methylation.tif]

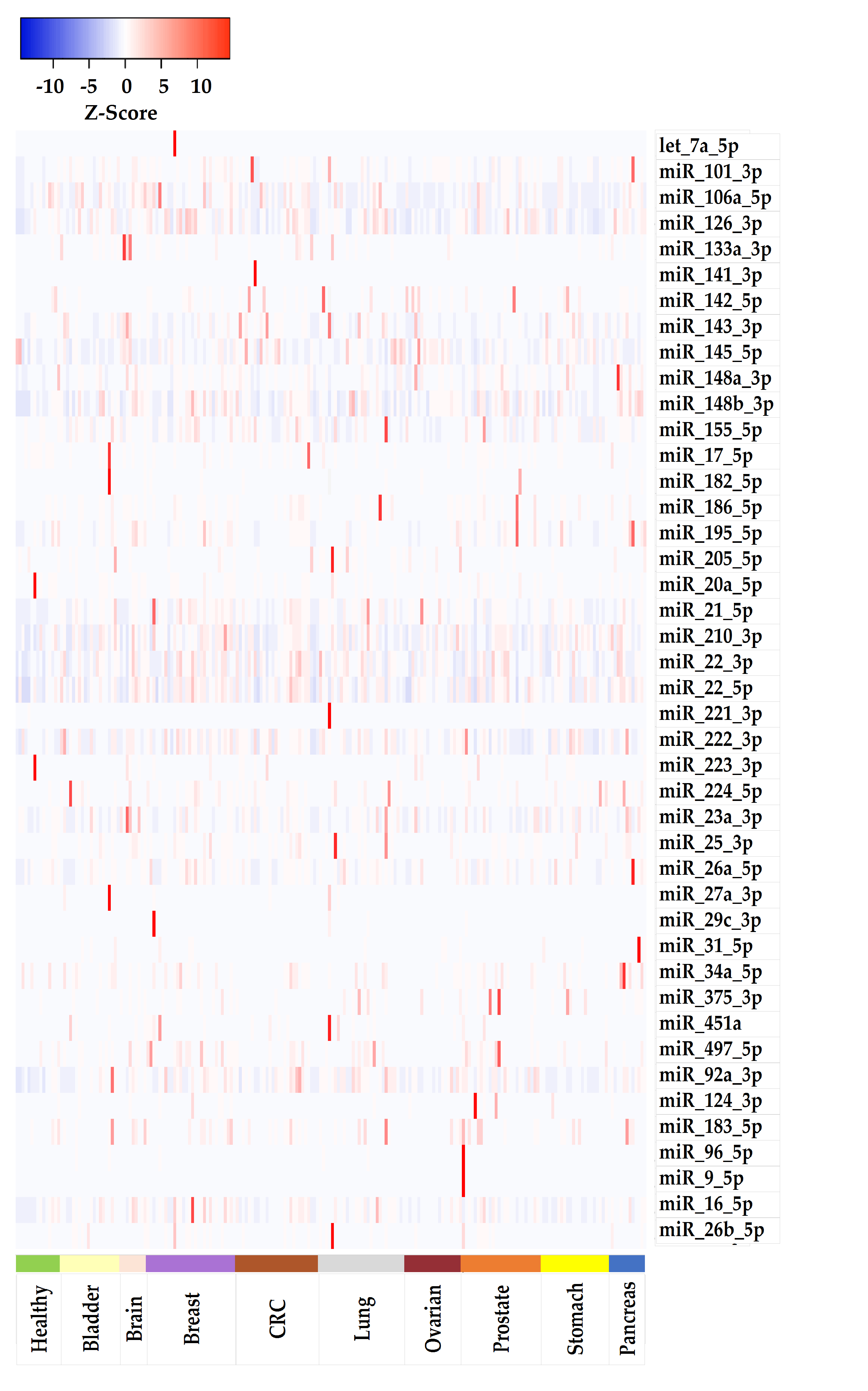

Supplement: Supplementary file 1 [file cancers-14-00462-s001.zip › cancers-1542395-supplementary/Supplementary Figure S4. Heatmap of the miRNAs levels.tif]
